# Pairwise correlation graphs from hippocampal population activity have highly non-random, low-dimensional clique topology

**DOI:** 10.1186/1471-2202-14-S1-P182

**Published:** 2013-07-08

**Authors:** Carina Curto, Chad Giusti, Keler Marku, Eva Pastalkova, Vladimir Itskov

**Affiliations:** 1 Department of Mathematics, University of Nebraska-Lincoln, Lincoln, NE, USA; 2 Janelia Farm Research Campus, HHMI, Ashburn, VA 20147, USA

## 

We analyzed the structure of cliques in pairwise correlation graphs obtained from population activity in hippocampus under a variety of behavioral conditions: open field exploration, wheel running, and sleep. Using topological data analysis, we found these graphs to be highly non-random by comparing their clique structure to that of Erdös-Rényi random graphs with matching edge probabilities. The clique topology in all three conditions was low-dimensional and consistent with what is theoretically expected from place cells during spatial navigation. Remarkably, the same pattern was also observed in the sleep data. To better understand how this correlation structure might arise, we considered a recurrent network with a simple Hebbian learning rule. We found the same clique structure emerged in the network when recurrent connections were formed in the presence of random sparse patterns of activity. This may provide a generic mechanism explaining how low-dimensional clique topology arises in hippocampal data.

Pairwise correlations are an important tool for understanding neuronal population activity [1]. Pairwise correlation graphs, where the edges reflect high levels of correlation between neurons, are often used as a proxy for underlying network connectivity. In this work, we are motivated by the question: *What is the structure of pairwise correlations in hippocampal population activity*? To address this question, we analyzed graded families of pairwise correlation graphs, parametrized by the threshold on correlation strength used to define the edges. In brain areas with receptive fields or place fields, the structure of the neural code has strong implications for structure of cliques in these graphs. This can be detected by examining the *clique topology *of the graph (specifically, topological invariants called 'Betti numbers' of the clique complex), and is closely tied to the dimension and topology of the underlying space [2]. For example, for hippocampal place cell activity during spatial exploration, pairwise correlation graphs are expected to have highly non-random and low-dimensional clique topology, due to the arrangement of place fields in a low-dimensional environment.

To test this hypothesis, we analyzed pairwise correlations graphs inferred from hippocampal CA1 data under a variety of behavioral conditions (open field exploration, wheel running, and sleep). We found that pairwise correlation graphs are (1) highly non-random, in a way that is easily detected by the clique topology; and (2) the clique topology is low-dimensional and consistent with what is expected of neurons firing according to place fields.

*Remarkably*, the sleep data showed a similar pattern, despite the fact that place fields are not observed under these conditions. This led us to ask: *How might low-dimensional clique topology arise in a highly plastic network, like hippocampus, during non-spatial behaviors? *To answer this question, we investigated a simple recurrent network, where pairwise connections are strengthened by Hebbian learning in the presence of sparse random patterns of externally driven activity. We found that the resulting network had highly non-random and low-dimensional clique topology, similar to what was observed in hippocampal data. This suggests that low-dimensional clique structure in pairwise correlation graphs may naturally emerge in networks with Hebbian plasticity.
